# Impacts of Green Tea on Joint and Skeletal Muscle Health: Prospects of Translational Nutrition

**DOI:** 10.3390/antiox9111050

**Published:** 2020-10-28

**Authors:** Hui-Ying Luk, Casey Appell, Ming-Chien Chyu, Chung-Hwan Chen, Chien-Yuan Wang, Rong-Sen Yang, Chwan-Li Shen

**Affiliations:** 1 Department of Kinesiology and Sport Management, Texas Tech University, Lubbock, TX 79409, USA; Huiying.Luk@ttu.edu (H.-Y.L.); Casey.Appell@ttu.edu (C.A.); 2 Center of Excellence for Integrative Health, Texas Tech University Health Sciences Center, Lubbock, TX 79430, USA; m.chyu@ttu.edu; 3 Healthcare Engineering Graduate Program, Texas Tech University, Lubbock, TX 79409, USA; 4 Department of Orthopedics and Orthopedic Research Center, Kaohsiung Municipal Ta-Tung Hospital and Kaohsiung Medical University Hospital, College of Medicine, Regeneration Medicine and Cell Therapy Research Center, Kaohsiung Medical University, Kaohsiung 800, Taiwan; hwan@kmu.edu.tw; 5 Department of Orthopedics, China Medical University Hsinchu Hospital, Zhubei City, Hsinchu 302, Taiwan; d30138@mail.cmuhch.org.tw; 6 College of Medicine, China Medical University, Taichung 400, Taiwan; 7 Department of Orthopedics, National Taiwan University Hospital, Taipei 100, Taiwan; rsyang@ntuh.gov.tw; 8 Department of Pathology Texas Tech University Health Sciences Center, Lubbock, TX 79430, USA

**Keywords:** green tea extract, osteoarthritis, sarcopenia, antioxidant, inflammation, mitochondria, autophagy, aging

## Abstract

Osteoarthritis and sarcopenia are two major joint and skeletal muscle diseases prevalent during aging. Osteoarthritis is a multifactorial progressive degenerative and inflammatory disorder of articular cartilage. Cartilage protection and pain management are the two most important strategies in the management of osteoarthritis. Sarcopenia, a condition of loss of muscle mass and strength, is associated with impaired neuromuscular innervation, the transition of skeletal muscle fiber type, and reduced muscle regenerative capacity. Management of sarcopenia requires addressing both skeletal muscle quantity and quality. Emerging evidence suggests that green tea catechins play an important role in maintaining healthy joints and skeletal muscle. This review covers (i) the prevalence and etiology of osteoarthritis and sarcopenia, such as excessive inflammation and oxidative stress, mitochondrial dysfunction, and reduced autophagy; (ii) the effects of green tea catechins on joint health by downregulating inflammatory signaling mediators, upregulating anabolic mediators, and modulating miRNAs expression, resulting in reduced chondrocyte death, collagen degradation, and cartilage protection; (iii) the effects of green tea catechins on skeletal muscle health via maintaining a dynamic balance between protein synthesis and degradation and boosting the synthesis of mitochondrial energy metabolism, resulting in favorable muscle homeostasis and mitigation of muscle atrophy with aging; and (iv) the current study limitations and future research directions.

## 1. Introduction

### 1.1. Prevalence of Osteoarthritis and Sarcopenia

Osteoarthritis (OA) and sarcopenia (SC), two major aging-related joint and skeletal muscle diseases, are prevalent among the elderly population and interact closely during the complex biological process of aging. Knee OA, a progressive joint disease characterized by the degeneration and inflammation of joints [1,2], is among the five leading causes of disability [3]. Aging is considered to be a major risk factor of OA as half of the world’s population 65 and older suffer from OA. Around 70 million Americans are at risk of OA and the financial expenditure for OA care is estimated to be $15.5–$28.6 billion per year in 2030 [3]. 

Sarcopenia, a progressive neuromuscular disorder causing a reduction in skeletal muscle mass and strength in the elderly, is associated with morbidity, metabolic dysregulation, functional disability, poor daily activity function, and mortality [4,5,6]. In general, a progressive and generalized deterioration of muscle mass starts at 25 years of age and results in an average of 30% muscle mass loss by the age of 80 [7]. The prevalence of SC is 5% to 13% in the population aged 60–70 years and 11–50% in that >80 years [8]. According to the World Health Organization, by 2050, about two billion people worldwide will be 60 years or older and approximately 400 million will be 80 years or older [9], making SC a potentially serious health issue. 

The pervasiveness and burden of knee OA pain relief and SC-associated disorders have significantly increased Medicare expenditures [10]. Therefore, alleviating the pain and mitigating pain-related dysfunction in patients with knee OA as well as slowing the process of SC-associated disorders are public health priorities.

### 1.2. Etiology of Osteoarthritis and Sarcopenia

A potential link may exist between OA and SC, the two most common musculoskeletal disorders in the elderly, through a connection of bone morphogenetic proteins and myostatin pathways, based on a few clinical and morphometric studies [11]. The common etiology for both OA and SC is described below, including excessive inflammation and oxidative stress, mitochondrial dysfunction, and reduced autophagy.

#### 1.2.1. Osteoarthritis

OA is a multifactorial progressive degenerative and inflammatory disorder of articular cartilage [12,13]. Although the exact mechanisms of OA pathogenesis remain unclear, overloading, trauma, an imbalance in inflammatory, and anti-inflammatory pathways all contribute to the impairment of cartilage structure and function [13,14,15]. Risk factors of OA include both systemic factors (aging, gender, obesity, inactivity, genetics, etc.) and mechanical factors (obesity, deranged joint morphology and alignment, overloading, muscle weakness, injury, etc.) [16]. 

Many inflammatory mediators accompany the progression of OA, including the activation of mitogen-activated protein kinases (mitogen-activated protein kinases (MAPKs), i.e., p38-MAPK, extracellular receptor kinases (ERK), and c-Jun N-terminal kinases (JNK)), transcription factors (nuclear factor-κB (NF-κB), and activator protein 1 (AP-1)) in chondrocytes and/or synoviocytes. The overexpression of inflammatory mediators (tumor necrosis factor-α (TNF-α), interleukin (IL)-1β, IL-6, IL-8, MMPs, cyclooxygenase-2 (COX-2), inducible nitric oxide synthase (iNOS), etc.) [17,18] stimulates the release of fibronectin and hyaluronan from the cartilage matrix [19] and induces excessive oxidative stress that may suppress complex I in the mitochondrial respiratory chain, resulting in the mitochondrial dysfunction of chondrocytes [20]. Excessive free radicals, such as nitric oxide, free oxygen species, and superoxide anions released by macrophages, further insult chondrocytes and induce cell death [20]. 

Emerging evidence suggests that the enhancement of autophagy in chondrocytes can delay the progression of OA by affecting intracellular metabolic activity [21,22,23,24]. In OA, autophagic activity was suppressed from the upstream initiation signaling (decreased in Unc-51–like kinase 1), phagophore initiation (decreased in Beclin 1), to downstream phagophore elongation (microtubule-associated protein 1 light chain 3) [21,23]. Cartilage-specific deletion of mTOR upregulated autophagy and protected mice from OA [25]. Similarly, autophagy activated by rapamycin (an inhibitor of mTOR) reduced the severity of experimental OA [26]. Accumulating evidence suggests that restoration of autophagy in senescent chondrocyte can refresh the vitality of chondrocytes and may be an effective therapeutic approach for OA. Altogether, inflammation, mitochondrial dysfunction, oxidative stress, and reduced autophagy in chondrocytes all impair function and promote catabolic processes of chondrocyte senescence and death [27]. Therefore, proper management of inflammation seems to be important for restoring chondrocyte function and integrity, pain relief, and thus controls the progress of OA.

#### 1.2.2. Sarcopenia

The reduction in muscle mass in SC could be the result of dysregulated protein degradation of existing muscles or satellite cell (muscle stem cell) dysfunction (impaired muscle regeneration and muscle growth) [28]. Several aging mechanisms have been identified contributing to the development of SC [29]. Risk factors such as excessive oxidative stress and chronic inflammation could result in cellular dysfunction [30]. Oxidative-stress-induced and chronic-inflammation-induced mitochondrial and satellite cell dysfunction has been shown to increase the accumulation of misfolded or damaged proteins and thus reduce cellular quality, promote cell senescence, and reduce muscle regeneration capacity, which could be attributed to SC [31]. In healthy cells, damaged or misfolded cellular protein is cleared via the proteolytic pathway (autophagy and mitophagy), which have shown to be compromised in aged cells [32].

A reduction in antioxidant cellular defenses (i.e., decrease in antioxidant enzymatic activity, reduction in the dysfunctional protein clearance, etc.) could result in ROS accumulation. Excess ROS could in turn damage the mitochondria and cause electron leakage from the electron transport chain, resulting in mitochondrial dysfunction [33]. This, at least partially, could elicit cellular senescence inflammation (IL-1β, IL-6, and TNF-α) [34]. Chronic low-grade inflammation increases NF-κB signaling, which induces atrogene-mediated protein degradation in the skeletal muscle [35] and results in muscle fiber damage or death and increasing ROS levels in circulation [36]. In addition, mitochondrial dysfunction has been shown to impair satellite cells’ activation and proliferation, which can impair muscle regeneration and, potentially, muscle growth [37]. 

Progressive cell death and/or increased protein degradation in mitochondria and skeletal muscle during aging can decrease lean mass over time [30,38]. Previous reviews have elucidated the importance of autophagy-mediated SC [32,39,40,41]. The activation of autophagy mediates suppression of the loss of skeletal muscle through signaling pathways, including phosphatidylinositol-3-kinase (PI3K), myostatin, and proteasome pathways [39]. Autophagy can promote either cell survival or cell death depending on the severity of ROS [42]. In cancer cachexia and autophagy knockout models, excessive or defective autophagic activity has been shown to reduce skeletal muscle mass [43,44]. Given that autophagy is critical in maintaining muscle mass and that autophagy activity decreases in aging muscle cells [32], it is plausible that aging cells require an autophagy stimulus to prevent the loss of muscle mass [45]. Similarly, mitophagy, a selective mechanism for removing dysfunctional mitochondria, is critical in maintaining the quality of mitochondria [46] and mitophagy is an emerging attributing factor toward the onset of SC [47,48,49]. An age-related decline in both autophagy and mitophagy activities may result in the loss of muscle mass and reduced satellite cells’ myogenic activities, which can potentially be reversed by increasing basal autophagy [50,51]. Therefore, attenuation of the oxidative and inflammatory stress and improvement of autophagy and mitophagy activities in skeletal muscle and satellite cells are crucial to skeletal muscle health. 

Recent evidence shows that both OA and SC result from excessive inflammation and oxidative stress, mitochondrial dysfunction, and the impairment of autophagy. Therefore, proper control of inflammation and oxidative stress can be a promising therapeutic approach for the management of both OA and SC. Currently, nonsteroidal anti-inflammatory drugs (NSAIDs) and acetaminophen are commonly used to treat knee OA pain; however, they may cause serious adverse side effects, especially among the elderly [52,53]. The most common intervention for SC treatment is exercise [54]. Other interventions for SC, such as hormone replacement therapy, angiotensin-converting enzyme inhibitors, and creatinine supplementation aimed to protect muscle loss, have their own disadvantages and certain toxic effects [55]. 

### 1.3. Green Tea

Since OA and SC are age-related and progressive, the early initiation of life-long treatment besides symptom control alone is urgent in maintaining joint and skeletal muscle health. Inflammatory mediators and their associated cellular events for both disorders seem to be practical targets. A growing body of evidence indicates that more and more elderly patients benefit from complementary and integrative medicine, such as nutraceuticals, to mitigate pain, maintain joint mobility, and improve physical functional capacity [56] for OA and attenuate muscle mass loss and strength for SC. 

Among different forms of nutraceuticals, teas (such as green tea, black tea, and Yerba Mate tea) have gained significant attention due to their health benefits. The main differences among different tea varieties are in their processing and chemical composition [57], as shown in Table 1. Compared to oxidized black tea and semioxidized Yerba Mate tea, green tea is nonoxidized with potent antioxidant, anti-inflammatory, and antioxidative properties that can alleviate pain progression and physical dysfunction in controlling the progression of both OA and SC. 

Tea (Camellia sinensis), especially green tea, which has catechins as the major active ingredient (12–24% dry weight), contains a large number of phenolic hydroxyl groups (-OH). The four monomers of catechins include epigallocatechin gallate (EGCG), epicatechin (EC), epigallocatechin (EGC), and epicatechin gallate (ECG). Green tea and its major bioactive component of the polyphenolic fraction of green tea, EGCG, have been suggested to be capable of protecting against cartilage loss and reducing the progression of OA in the past decade. Recently, green tea catechins (GTCs) demonstrated the potential to re-establish homeostasis of skeletal muscle cells and even attenuate muscle mass loss. In this review, we summarize the state of knowledge of laboratory preclinical research and limited human studies assessing the effects of green tea and EGCG on joint and skeletal muscle, along with a discussion of future directions in translational research. 

## 2. Beneficial Effects of Green Tea Extract (GTE) on Joint Health

### 2.1. Cell and Tissue Explant Studies

Table 2 summarizes the effects of green tea and EGCG on joint health based on in vitro (cell and tissue explant), in vivo (animal), and human studies. Adcocks et al. first reported that EGCG, rich in antioxidant capacity, protects the cartilage matrix (proteoglycan and type II collagen) from degradation in bovine cartilage explants and human OA cartilage explants [58]. In follow-up studies, other authors corroborated that EGCG reduced the release of glycosaminoglycans (GAG, a component of cartilage matrix) and type II collagen from human cartilage explants [59,60] and selectively inhibits the A disintegrin and metalloproteinase with thrombospondin motifs (ADAMTS)1, ADAMTS4, and ADAMTS5 [61,62]. The chondroprotective effects of EGCG are due to EGCG’s anti-inflammatory action, as evidenced by inhibiting the expression of proinflammatory genes (i.e., COX-2, TNF-α, MMP-1, MMP-13, iNOS, and ADAMTS5) [59,62,63,64,65,66,67,68,69]. Such anti-inflammatory effects of EGCG are mediated by the suppression of NF-κB signaling cascades [59,63,66,69,70,71,72] and MAPK including p38-MAPK, Erk1/Erk2 [63,65,71,73], activator protein-1 (AP-1) [59,70], c-jun *N*-terminal kinase (JNK) activation, and IKKβ kinases phosphorylation [59,64,73] in chondrocytes as a result of the decreased differentiation of chondrocytes [74]. With respect to synoviocytes, Zheng et al. recently reported that EGCG-glucosamine nanoparticles exhibit antiarthritic activity in human-fibroblast-like synoviocytes-OA cells due to EGCG’s anti-inflammatory action [75]. Furthermore, EGCG has been used to stabilize an osteochondral xenograft prior to graft implantation for the repair of a defect [76]. Elder et al. also demonstrated that EGCG treatment resisted an osteochondral xenograft from collagenase degradation via increased collagen crosslinking as a result of the restored mechanical properties of osteochondral xenograft [76]. 

In addition to EGCG’s anticatabolic effects on OA, EGCG also has anabolic effects on OA. Andriamanalijaona et al. reported that EGCG stimulated the IL-1β-induced expression of transforming growth factor (TGF)-β1, TGF-β2, and its receptors TGF-βR1 and TGF-βRII in bovine articular chondrocytes, resulting in enhanced type II collagen and aggrecan core protein synthesis in human articular chondrocytes [63]. Huang et al. showed EGCG supplementation stimulates chondrocyte growth and the synthesis of cartilage extracellular matrix by enhancing the gene expression of aggrecan, collagen type II, and SRY-Box Transcription Factor (SOX9) and suppressing the gene expression of collagen in primary rabbit articular chondrocytes [74]. Jin et al. recently demonstrated that EGCG addition to hyaluronic-acid-based hydrogel synergistically stimulates chondrogenic regeneration by increasing the gene expression of collagen type II, SOX9, and aggrecan in primary porcine 3D encapsulated OA chondrocytes [62]. 

Table 3 summarizes the effects of ECGC on selected miRNA expressions in human or murine chondrocytes. MicroRNAs (miRNAs) are endogenous and noncoding single-stranded RNAs with a profound role in gene regulation at post-transcriptional levels [77]. miRNA (also named miR) regulation and expression have become an emerging field in determining the mechanisms involved in a variety of inflammation-mediated diseases, including OA. In OA, specific miRNAs have been identified and linked to OA risk factors, such as inflammation, obesity, autophagy, and imbalanced cartilage homeostasis [77]. miRNAs are able to modulate most OA relevant genes in stimulated human OA chondrocytes [59,67,78,79,80,81,82,83,84,85]. For example, miR-27b is a direct regulator of MMP-13 in human OA chondrocytes [78]. hsa-miR-26a-6p regulates the expression of iNOS via the activation of NF-κB signaling pathways [83]. Furthermore, miRNAs including miR-27b [79], miR-26a-5p [80], hsa-miR-199a-3p [67], miR-127-5p [81], miR-602 [82], miR-608 [82], miR-320 [83], miR-558 [84], miR-9 [85], and miR-381 [86] also play significant roles in regulating key inflammatory genes in the development of OA. Rasheed et al. first demonstrated that EGCG inhibits COX-2 mRNA/protein expression by upregulating miRNA hsa-miR-199a-3p expression in IL-1β-induced human OA chondrocytes [67]. In a study on a human miRNA microarray of 1347 miRNAs, EGCG upregulated the expressions of 19 of them (such as hsa-miR-140-3p) and downregulated the expressions of 17, whereas the expressions of the remaining miRNAs remained unchanged in IL-1β-induced human OA chondrocytes [87]. The IL-1β-induced expression of ADAMTS5 correlated with the downregulation of hsa-miR-140-3p, and EGCG-induced coregulation between ADAMTS5 and hsa-miR-140-3p became reversed in OA chondrocytes transfected with anti-miR-140-3p [87]. Zhang et al. recently reported that the pretreatment of green tea polyphenols mitigates lipopolysaccharide-induced inflammatory response along with suppression of MAPK and NF-κB pathways by positively regulating miR-9 (an anti-inflammatory regulator) expression in murine chondrogenic ATDC5 cells [71]. These findings suggest that the chondroprotective roles of green tea and EGCG may be associated with EGCG’s ability to suppress inflammatory response by modulating miRNAs expression [87]. 

### 2.2. Animal Studies

Green tea and EGCG have been shown to protect cartilage degradation in a variety of animal OA models. In a study using a mouse model with collagen-induced arthritis, Haqqi et al. first reported that the supplementation of green tea polyphenols in the drinking water significantly reduced the incidence of arthritis in mice, as shown by (i) decreased type II collagen-specific IgG levels, (ii) the reduced expression of proinflammatory genes (COX2, TNF-α, IFN-γ), (iii) decreased joint infiltration by TNF-α and IFN-γ-producing cells, and (iv) increased neutral endopeptidase activity in arthritic joints [88]. In a study using a mouse model with intra-articular carrageenan-induced OA, compared with control, the GTE-treated group demonstrated lower levels of lipid peroxides, NO, and total thiols in the plasma, as well as reduced inflammatory cells infiltrating the synovial membrane [89]. In a study of mice with post-traumatic OA by the destabilization of the medial meniscus (DMM), Leong et al. reported that EGCG significantly slows progression in early- and midstage OA development, as indicated by lower OARSI scores and higher locomotor behavior [90]. EGCG-treated post-traumatic OA mice exhibited a reduced degradation of both type II collagen and aggrecan in the articular cartilage matrix by suppressing the gene expression of MMP-1, -3, -8, and -13, ADAMTS5, IL-1β, and TNF-α, and inducing the gene expression of MMP-repressing transcriptional regulator CITED2 in the articular cartilage [90]. Recently, in a study using a mouse model with surgically induced OA, Jin et al. reported that treatment with crosslinking of EGCG into hyaluronic acid (a major component of the cartilage extracellular matrix) hydrogel protects cartilage from physical abrasion in arthritic joints, as demonstrated by mitigated loss of glycosaminoglycan and type II collagen as well as reduced expression of collagen I and X [62].

### 2.3. Human Studies

Although the existing evidence based on cell, tissue explants, and animal studies suggests that green tea and EGCG could mitigate cartilage degradation by modulating multiple targets in joint during the OA progression, there are only limited studies to test its antiarthritic effects in humans. In a five-year Murakami cohort study, Takiguchi et al. determined lifestyle-related modifiable factors, including age, BMI, metabolic equivalent of task (MET) score, smoking, and green tea consumption, of symptomatic knee OA in an East Asian population [18]. In men (not in women), older age (≥60 years), higher BMI, higher MET score, and lower green tea consumption were significantly associated with an incidence of knee OA as assessed by the Kellgren–Lawrence scale. The endogenous sex hormone and estrogen in women might have attenuated the possible anti-inflammatory effects of GTE due to a ceiling effect compared to men [91]. In a randomized open-label active-controlled clinical trial of 55 OA adults, Hashempur et al. reported that compared to the control group receiving diclofenac, the intervention group receiving GTE plus diclofenac for four weeks had reduced pain scores and improved total Western Ontario and McMaster Universities Osteoarthritis Index (WOMAC) and WOMAC-physical scores, but no change in scores of WOMAC-pain and WOMAC-stiffness [92].

## 3. Beneficial Effects of GTE on Skeletal Muscle Health

### 3.1. Effect of GTE on Oxidative Damage of Skeletal Muscle Cell Culture Studies

#### 3.1.1. In Vitro Studies

Table 4 summarizes the effects of green tea and EGCG on skeletal muscle health based on in vitro (cell and tissue explant), in vivo (animal), and human studies. In an in vitro model, incubating C2C12 with 50 μM of EGCG showed decreased ROS and increased anti-ROS enzyme activity (i.e., catalase (CAT) and glutathione peroxidase (GSH-Px)) [93]. Furthermore, incubating ROS-pretreated primary muscle cells with 20 μM of green tea extract (GTE) for 48 h demonstrated an increase in glutathione peroxidase content and cell viability [94]. Similarly, Babu et al. incubated C2C12 pretreated with GTE (40–160 μM) with 100 μM citrinin (ROS inducer), which showed a suppression of the lactate dehydrogenases (an indication of membrane stability and decreased cytotoxicity), an increase in cell viability, and the protection of myotube from ROS-induced damage [95]. These results suggest that EGCG or GTE supplementation could be a treatment or a preventative agent of SC-induced ROS, at least at the molecular level. 

#### 3.1.2. Rodent Studies

The use of GTE has been shown to improve oxidative state and skeletal muscle health (e.g., strength, morphological integrity, and the number of muscle stem cells). For example, in a dystrophic C57BL/10-mdx (mdx) model, the abnormally high ROS results in severe muscle damage and affects muscle integrity (i.e., membrane stability) [96]. Administering EGCG (5 mg/kg, 4×/week for eight weeks) resulted in less fibrosis and fewer necrotic myofibers in mice with muscular dystrophy [97]. Similarly, supplementing the diet with 0.01% to 0.25% of GTE for 4–5 weeks increased the antioxidant potential in the plasma [98] and reduced necrosis in the extensor digitorum longus (EDL) muscles of mdx mice [98,99]. Moreover, EGCG supplementation decreased protein carbonyl content, a marker of oxidative stress, in aged (34 months) male albino Wistar rats [100]. The findings that GTE supplementation enhances total antioxidant potential (i.e., GSH-Px, SOD, CAT) and lowers carbonylated protein levels in skeletal muscle are consistent in a variety of rodent models including BALB/c mice, female Sprague–Dawley rats, and male Kunming mice [101,102,103]. Interestingly, in male ICR mice, tannase-converted GTE (80 μM) with greater antioxidant capacity increased antioxidant enzyme SOD and CAT content [104]. 

#### 3.1.3. Human Studies

In male soccer players and sprinters, GTE supplementation (450 mg/d and 980 mg/d) for four to six weeks resulted in an increase in systemic total antioxidant capacity and a decrease in MDA (a marker for oxidative stress) [105,106]. In response to a bout of exercise, Panza et al. showed that a week of GTC supplementation (2 g of leaves in 200 mL of water) three times per day prior to a single bout of resistance exercise protected against the systemic oxidative exercise-induced damage in weight-trained men [107]. Furthermore, combining GTE (250 mg/d) supplementation with endurance training for four weeks increased systemic total antioxidant capacity and reduced MDA production but not with GTE supplementation or endurance training alone [108]. However, Silva et al. reported a change in only creatine kinase levels following a 15-day GTE supplementation in untrained men [109]. These results from human studies suggest that GTE supplementation may not affect the basal oxidative state but may reduce exercise-induced oxidative stress. 

### 3.2. Effect of GTE on Inflammation of Skeletal Muscle Animal studies

GTE has been shown to reduce proinflammatory cytokines in skeletal muscle with elevated inflammatory signaling. Female Sprague–Dawley rats fed a high-fat diet (aimed to induce obesity) supplemented with GTE (0.5% *wt/vol* GTE for 12 weeks) showed lower serum concentrations of IL-1α, IL-2, GM-CSF, IFN-γ, and TNF-α compared to a high-fat diet without GTE [102]. Similarly, male Wistar rats fed a high-fat diet supplemented with GTE (1 g/kg GTE for six weeks) had a lower intramuscular TNF-α and COX-2 RNA expression than a high-fat diet alone [110]. Similar results were reported in studies using the RNAseq technique, where inflammation-related genes (Cd163, Cfh, Il33, C3, Hp, Lbp) were downregulated in C57bL/6J mice fed a high-fat diet supplemented with GTE (5% *wt/wt* GTE for eight weeks) than those fed a high-fat diet alone, and the addictive effect of exercise on GTE reduced inflammation [111]. Mice with obesity induced by a high-fat diet treated with endurance training (15 min of treadmill running 6×/week for eight weeks) along with GTE supplementation showed a greater decrease in inflammation-related genes (i.e., Cd163, Cfh, Il33, C3, Hp, Lbp) than those treated with GTE or endurance exercise alone [111]. In addition, male ICR mice supplemented with GTE (0.5% *wt/wt* for three weeks) prior to a bout of exercise causing muscle damage showed a lower TNF-α, IL-1b, and MCP-1 RNA expression than mice with exercise-induced muscle damage alone [112]. To further investigate whether the decrease in inflammatory cytokines is regulated by the upstream regulator (i.e., NF-κB), Zhang et al. reported an increase in phosphorylated IkBα (NF-κB inhibitor) expression, which, at least in part, explained their observation of decreased inflammatory-related genes in mice fed a high-fat diet supplemented with GTE [111]. Such results are supported by a decreased intramuscular NF-κB protein content in male C57BL/6 mice with LLC cells tumor treated with GTE supplementation at 0.6 mg/mouse for 12 days [113] and mdx mice treated with GTE supplementation [114]. These results have consistently demonstrated the anti-inflammatory effect of GTE via upstream regulation, albeit they are limited to rodent models. In the future, a human study is needed to further elucidate the anti-inflammatory properties of GTE, considering chronic low-grade inflammation is a major attributing factor for the development of sarcopenia.

### 3.3. Effect of GTE on Autophagy in Skeletal Muscle

Autophagy promotes a muscle mass increase in SC muscle models [28]. A loss in skeletal muscle mass during aging could be attributed to the intracellular accumulation of damaged proteins and organelles as a result of reduced autophagic activity [115]. Emerging evidence suggests that GTE supplementation may reverse the suppression of autophagy signaling in aged skeletal muscles [51,116,117]. In in vitro studies, GTE initiated upstream signaling (increased FoxO3 nuclear accumulation and AMPK activity) [118] and phagophore initiation (i.e., decreased inhibit beclin-1 inhibitor) [119] for autophagy. GTE supplementation (polyphenol blend; 40% catechins, 3–8% EGCG) for 28 days suppressed an increase in Bcl-2 (beclin-1 inhibitor) and BAD (autophagy inhibitor) due to muscle damage in men [120]. It is noteworthy that incubating C2C12 muscle cells with 25 μM of EGCG and 300 μM of H_2_O_2_ for 48 h resulted in a reversion of AMPK activity compared to a decreased AMPK activity with EGCG treatment alone [93]. Wang et al. suggest that in healthy muscle cells, EGCG effectively reduces ROS such that the induction of autophagy may not be warranted. However, in muscle cells containing excessive ROS (i.e., in SC muscle), the antioxidative properties of EGCG alone may not be sufficient to reduce the ROS burden, requiring increased autophagy activity [93]. On the other hand, Mirza et al. reported an overall decrease in protein degradation rates following EGCG (10 μM) treatment [121]. Future studies are required to fully elucidate the effect of GTE on autophagy in SC muscle models.

Maintaining mitochondrial quality is linked to skeletal muscle health [122] and improved exercise tolerance [123]. Mice treated with EGCG had an increased expression of mitophagy (PINK1, UCP2) and autophagy (LC3B:LC3A, ATG16L, DAPK, TM9SF1, and PIM2) in skeletal muscle [45,124], but other studies showed inconsistent results [125,126]. EGCG supplementation was shown to mitigate the hindlimb suspension-induced decrease in mitophagy [45]. Takahashi et al. reported that in the absence of exercise, GTE supplementation might help mitigate the age-related reduction in autophagic activity [45]. Furthermore, supplementing GTE prior to exercise could potentially help reduce the negative effect of exercise-induced oxidative stress in the older population [45]. Although no study has directly examined the effect of EGCG on the crosstalk between ROS content, autophagy, and mitophagy activity related to skeletal muscle heath, published results linking the increase in autophagy to improved muscle mass suggest that EGCG could delay muscle mass loss in SC-like rats [127], the attenuation of skeletal muscle mass loss in mice with Lewis lung carcinoma cachexia [98], and enhance muscle function in mice with Duchenne muscular dystrophy [113]. This may, at least in part, be related to EGCG-induced autophagy of skeletal muscle. 

### 3.4. Effect of GTE on Mitochondria-Related Metabolism in Skeletal Muscle

Besides regulating mitochondrial quality, increasing the generation of mitochondria (mitochondria biogenesis) is also an important factor for skeletal muscle health and improving exercise capacity. Mitochondrial biogenesis via peroxisome-proliferator-activated receptor gamma coactivator-1 alpha (PGC-1α) signaling is one of the most important molecular adaptations from endurance exercise. In rodent studies, GTE supplementation with endurance exercise increased PGC-1α mRNA expression compared to GTE supplementation or endurance exercise alone [128,129]. Incubating C2C12 muscle cells with EGCG (25 μM) for 48 h showed a decrease in PGC-1α protein content [93]. However, the relationship between GTE and mitochondrial biogenesis in skeletal muscle has not been comprehensively elucidated.

Metabolically, GTE supplementation has been shown to increase mitochondrial enzymes involved in fatty acid oxidation. Sae-tan et al. reported that EGCG supplemented in a high-fat diet for 16 weeks increased mRNA expression of medium-chain acyl coA decarboxylase (MCAD) involved in mitochondrial fatty acid β-oxidation in the skeletal muscle of obese mice [125]. The fatty acid metabolism of skeletal muscle appeared to be further improved by combined GTE supplementation and exercise [128,129]. For instance, Murase et al. reported that senescence-accelerated mice treated with GTE supplementation together with treadmill running for 10 weeks achieved a greater increase in skeletal muscle fatty acid β-oxidation than those treated with either the GTE supplement or treadmill running alone, as shown by an increase in mRNA levels of mitochondria-related metabolism molecules such as mitochondrial cytochrome b, mitochondrial cytochrome c oxidase II, III, and IV [129]. Sae-Tan et al. corroborated the above result that the combination of GTE supplement and exercise increased the expression of mitochondria-related metabolism molecules in skeletal muscle of obese mice compared to those treated with GTE supplement or exercise alone [128]. 

The GTE-induced improvement of mitochondrial capacity to metabolize fatty acid may also translate into enhanced exercise capacity. In both aging and obese rodent models, two to four weeks of endurance training (swimming and running) combined with GTE supplementation increased exercise time to exhaustion and the total distance covered compared to either GTE or treadmill running alone [130,131,132,133]. The improvement in exercise capacity was attributed to increased reliance on fatty acid metabolism including β-oxidation activity, plasma free fatty acid concentration, and fatty acid translocase [130,131]. In an aged mice model, supplementing GTE for 10 weeks maintained endurance capacity compared to young mice [129]. Besides endurance capacity, five weeks of GTE supplementation in the standard diet provided 30 to 50% greater residual force production in mdx mice than those without GTE, suggesting that GTE was able to mitigate loss in force production without exercise training [98]. The above results suggest that EGCG treatment contributes to the improvement of mitochondrial health and efficacy in rodent models. Similarly, in humans, GTE (500 mg/d for eight weeks) supplementation was shown to increase reliance on fat oxidation during 60 min cycling exercise at 75% VO2max [134].

### 3.5. Effect of GTE on Satellite Cells, Muscle Damage, and Recovery

Satellite cell, a skeletal muscle stem cell, is important for muscle regeneration and repair [135]. Age-related decline in satellite cell number (i.e., stem cell pool) and disrupted myogenic progression (activation, proliferation, and differentiation) are among many factors that cause a decline in muscle mass and muscle regeneration capacity [136]. Incubating C2C12 muscle cells with EGCG or GTE resulted in an increase in MyoD (indicator of proliferation) as well as Myf5 and myogenin (indicator of differentiation) gene expression [137,138]. Similarly, supplementing aged mice with tannase-converted GTE increased MyoD, Myf5, and myogenin and decreased myostatin (a protein that suppresses satellite cell proliferation and differentiation) expression in skeletal muscle development [104]. 

Morphologically, EGCG supplementation was linked to an increased myonuclei number, myotube formation, cross-sectional area, and muscle mass through in vitro and rodent model studies [114,127,139,140,141]. There was no difference in lean mass gain between EGCG (250 mg/kg) alone and EGCG running for 39 days in adult mice [142]. In a limb-immobilization model, aged rats were treated with a GTE supplement (50 mg/kg) for seven days prior to 14 days of hindlimb suspension in one group, and another group received the same treatment plus a subsequent 14 days of ambulation. Both groups demonstrated increased satellite cell activation and muscle cross-sectional area [143]. Although the above results represent strong evidence with regard to the potential benefits of GTE in SC myogenic progression and muscle regeneration, future human research in this area is warranted.

### 3.6. Possible Mechanisms Related to GTCs’ Effects on Skeletal Health

**Figure 1:**
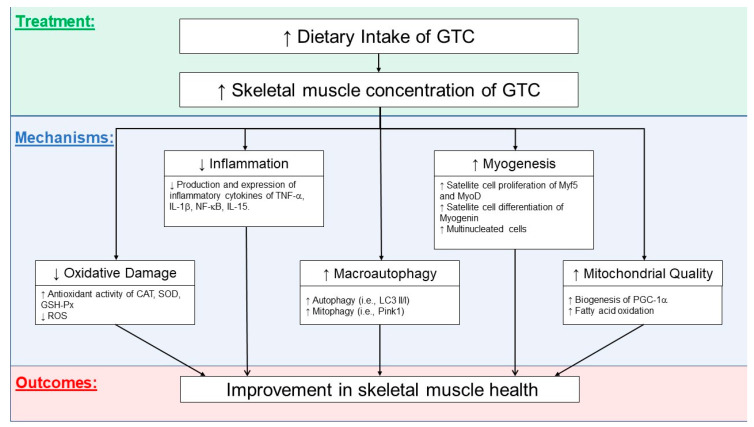
Possible mechanisms related to the effects of green tea catechins (GTCs) on an improvement in skeletal muscle. CAT, catalase; SOD, superoxide dismutase; GSH-Px, glutathione peroxidase; TNF-α, tumor necrosis factor-alpha; IL-1β, interleukin-1beta; IL-15, interleukin-15; NF-κB, nuclear factor kappa-light-chain-enhancer of activated B cells; LC3 II/I, microtubule-associated proteins 1B/1A light chain 3B; PINK1, PTEN-induced kinase 1; Myf5, myogenic factor 5; MyoD, myoblast determination protein 1; PGC-1α, peroxisome proliferator-activated receptor gamma coactivator 1-alpha.

Figure 1 illustrates the potential effects of GTCs on skeletal muscle. GTCs have been shown to effectively suppress inflammation and oxidative stress and promote autophagic and mitophagy activities. Improving the quality of mitochondria (decreased ROS leakage and improved oxidative metabolism) and potentially increasing mitochondrial biogenesis could provide machinery for muscle cell growth and regeneration capacity. Although these potential benefits of GTCs on skeletal muscle health need to be further elucidated, GTCs are promising dietary nutritional supplements capable of maintaining muscle homeostasis and combating disuse muscle atrophy with aging.

## 4. Potential Side Effects of Green Tea

Although existing evidence supports the beneficial effect of green tea on joint and muscle health, there are potential side effects associated with green tea intake. Since EGCG in GTP can bind with iron, excessive green tea drinking can lead to iron deficiency anemia [39,146]. Caffeine in green tea may interfere with some medications. The most serious caffeine-related central nervous system effects are seizure and delirium, followed by cardiovascular side effects such as tachycardia and cardiac arrhythmia. The polycyclic aromatic hydrocarbon-inducible cytochrome P450 (CYP) 1A2 participates in the metabolism of caffeine and many clinical drugs including antiarrhythmics (mexiletine), some selective serotonin reuptake inhibitors (particularly fluvoxamine), antipsychotics (clozapine), bronchodilators (furafylline and theophylline), and quinolones (Ciprofloxacin) [147]. Thus, pharmacokinetic interactions at the CYP1A2 enzyme level may cause toxic effects during concomitant administration of caffeine and the aforementioned drugs [148]. Green tea caffeine also inhibited the metabolism of clozapine, an antipsychotic drug, leading to clozapine toxicity [149]. Vitamin K in green tea inhibits the effects of warfarin, an anticoagulant [150]. Too much GTP may cause hypokalemia leading to muscle weakness [151]. Excessive green tea caffeine may impair thyroid function [152]. Caffeine in green tea is a nervous system stimulant. Consuming more than 300 mg of caffeine per day may reduce sleep quality and cause insomnia, irritability, depression, anger, and anxiety [153]. Too much green tea may cause heartburn, which is a typical symptom of gastroesophageal reflux disease [154]. The diuretic effect of caffeine affects bladder function by increasing neuronal activation of the micturition center [155]. Lastly, green tea may stain teeth [156].

## 5. Conclusions, Limitations, and Future Directions of Research

Both OA and SC are closely related to aging and are prevalent in the elderly population. This review summarizes the state of the knowledge about possible pharmacological mechanisms of green tea and EGCG in terms of mitigation of OA progression via its anti-inflammatory, antioxidative stress, and antioxidant properties in preclinical and clinical studies. GTCs and EGCG have been shown to (i) downregulate inflammatory mediators, COX-2, IL-1β, TNF-α, MMPs, iNOS, and ADMTS5, by suppressing NF-κB and MAPK pathways, (ii) upregulate anabolic mediators, TGF-β1, TGF-β2, and its receptors TGF-βR1 and TGF-βRII, and (iii) modulate miRNAs expression, resulting in reduced chondrocyte death, collagen degradation, and cartilage erosion. GTCs including EGCG have great potential as a novel approach in mitigating the progression of OA and SC (Figure 1).

There are some limitations to this review. Overall, this review is based on limited studies investigating the effects of GTCs on OA and SC during aging. Green tea contains different types of catechins, the composition of which varies with planting environments and seasons. Such a variation in ingredients of GTCs and the interaction among them may result in different benefits to joint and skeletal muscle health, although this limitation does not apply to a number of studies covered in this review using pure monomers of catechins such as EGCG. The translation of results from animal studies to humans requires the investigation of the bioavailability, safety, and efficacy of green tea and EGCG in OA or SC patients. As most clinical studies on GTCs’ effects on OA and SC are not randomized controlled clinical trials, current limited human studies do not provide evidence at a sufficiently high level to confirm GTCs’ beneficial effects on joint and skeletal muscle health in OA and SC patients. Optimal treatment, dosage, and time of GTCs’ intervention for the maintenance or promotion of joint and skeletal muscle health are important but not well studied yet. Quite a few studies covered in the present review included exercise intervention in addition to green tea supplement. Good lifestyles such as exercise may also improve joint and skeletal muscle health. However, there is limited knowledge on the interaction between different lifestyle factors and green tea supplements and how they benefit OA and SC patients.

For patients with concomitant OA and SC, the disorders may interact and complicate clinical progression. In this review, we found a common pathomechanism for both disorders. Therefore, a common therapeutic strategy can be employed for the management of the common denominators for both OA and SC, i.e., inflammation, excessive oxidative stress, mitochondrial dysfunction, and impairment of autophagy activity. Such a treatment may need to start early in life and last a lifetime, considering the progression of the disorders with the continuous and irreversible aging process. GTCs have been demonstrated as potential functional products to prevent, delay, alleviate, and even treat joint- and skeletal-muscle-related disorders related to aging. EGCG may be adjunctively administered with OA or SC drugs to reduce the drug dose and interval between episodes by additively or synergistically enhancing anti-OA and anti-SC efficacies. Well-designed prospective human clinical trials and translational studies to test pharmacological and nonpharmacological therapies are warranted to evaluate their effects on OA and SC in terms of biochemical outcomes, alleviation of symptomatic pain and muscle mass reduction, and improvement of physical function, especially in patients with concomitant OA and SC.

Gut microbiota in the gastrointestinal tract has been identified as a possible factor causing age-related cartilage and skeletal muscle disorders. Emerging evidence suggests that the gut microbiome plays an important role in joint health by modulating nutrient absorption, intestinal permeability, metabolic immunity, cartilage–gut microbiome axis, and excretion of functional metabolites [157,158,159,160]. The mechanisms concerning how intestinal microbiota composition and metabolites affect OA and SC pathogenesis remain unclear [161,162,163,164]. Studies have reported that long-term treatment with green tea polyphenols modified the gut-microbiota-dependent metabolism of energy, bile constituents, and micronutrients in female Sprague–Dawley rats [165,166]. Future research should investigate the potential impacts of green tea and EGCG on the gut microbiota composition and functionality along with joint parameters such as chondrocyte metabolism, cartilage integrity, and pain reduction in OA animals. Such studies can provide new knowledge on the effects of green tea and EGCG on joint health in terms of the modification of the gut microbiota. A similar approach can be taken to investigate the effects of green tea and EGCG on the gut microbiota composition and functionality and how they in turn affect skeletal muscle parameters, such as myocyte metabolism, skeletal muscle size, composition, and function in animals with SC. 

## Figures and Tables

**Table 1:** Comparison of tea leaves processing and chemical composition among green tea, black tea, and Yerba Mate tea [57].

	Green Tea	Black Tea	Yerba Mate Tea
Processing of tea leaves	Steamed or pan-fried; not oxidized.	Withered and fermented and not blanched before drying; oxidized.	Flash heated over an open flame; semioxidized.
Types of polyphenols	Caffeic acid, caffeine, catechin, coumaric acid, EGCG, EC, EGC, ECG, gallic acid, gallocatechin gallate, kaempferol, myricetin, quercetin, quinic acid, rutin.	Caffeine, catechin, catechin gallate, ECG, gallic acid, gallocatechin gallate, kaempferol, myricetin, procyanidin, quercetin, rutin, theaflavin, theobromine.	Caffeic acid, caffeine, caffeoyl derivatives, caffeoylshikimic acid, chlorogenic acid, feruloylquinic acid, kaempferol, quercetin, quinic acid, rutin, theobromine.

**Table 2:** Summary of studies on the effects of green tea and epigallocatechin gallate (EGCG) on joint health.

First Author, Year of Study, Citation	Experimental Design and Treatments	Effects of Green Tea or EGCG
***Cellular Studies***		
Adcocks, 2002, [58]	**Model**: Bovine cartilage explants cotreated with TNF-α (3 nM) **Treatments**: EGCG (0.2, 2, 20, or 200 µM) for 5 days **Model**: Human cartilage from an OA knee joint cotreated with IL-1β (3 nM), TNF-α (6 nM) **Treatment**: EGCG (20 µM) for 9 days	Compared to the control group, the EGCG groups showed: ↓ Proteoglycan and type II collagen degradation
Ahmed, 2002, [68]	**Model**: Human primary chondrocytes cotreated with IL-1β (5 ng/mL) **Treatments**: Control, EGCG (20, 50, 100, or 200 μM) for 24 h	Compared to the control group, the EGCG groups showed: ↓ iNOS expression and NO production ↓ COX-2 expression and PGE2 production ↓ LDH release
Ahmed, 2004, [59]	**Model**: Human primary chondrocytes or human cartilage explants cotreated with IL-1β (50 or 10 ng/mL) **Treatments**: EGCG (20, 50, 100, or 200 μM) for 24 or 72 h	Compared to the control group, the EGCG groups showed: ↓ mRNA and protein expression of MMP-1 and MMP-13 in chondrocytes ↓ GAG release from human cartilage explants ↓ Transcription activity of NF-κB and AP-1
Akhtar, 2011, [65]	**Model**: Human primary chondrocytes with IL-1β (5 ng/mL) after EGCG **Treatments**: Control, EGCG (10–100 μM) for 24 h	Compared to the IL-1β group, the IL-1β + EGCG groups showed: ↓ ENA-78, GM-CSF, GRO, GRO-α, IL-6, IL-8, MCP1, MCP-3, MIP-1β, GCP-2, MIP-3α, IP-10, NAP-2, LIF via ↓ activation of NF-κB and JNK-MAPK
Andriamanalijaona, 2005, [63]	**Model**: Bovine primary articular chondrocytes **Treatments**: Pretreated with EGCG (20 or 50 µM) for 24 h and then cotreated with by IL-1β (10 ng/mL) for 24 h	Compared to the control group, the EGCG groups showed: ↑ Type II collagen and aggrecan core protein expression ↑ mRNA expression of TGF-β1, TGF-β2, TGF-βR1, and TGF-βII ↓ mRNA levels of MMP-1, MMP-3, MMP-13, aggrecanase-1, aggrecanase-2, iNOS, COX-1, COX-2 (anti-inflammatory effect) ↓ MAPK (Erk1/Erk2, p38 kinase), NF-κB, and AP-1 activity
Bae, 2010a, [72]	**Model**: Primary human articular cartilage with or without OA treated with EGCG. Then, cartilage is implanted to cartilage defects in rabbits **Treatments**: Control (no EGCG) and EGCG (1 mM) at 4 °C for 1–4 weeks	Compared to the control group, the EGCG group showed: Preserving and repairing articular cartilage by reversibly regulating cell cycle at G0/G1 phase and NF-κB expression
Bae, 2010b, [60]	**Model**: Human articular cartilage **Treatments**: Control (no RGCG), cotreated EGCG (1 mM), and pretreated EGCG for 2 weeks	Compared to the control group, the EGCG group showed: ↑ GAG content and total collagen ↓ Denaturation of extracellular matrix
Elder, 2017, [76]	**Model**: Porcine osteochondral xenograft **Treatments**: Control (no EGCG), EGCG at 0.04%, 0.2%, and 1% *w/v*	Compared to the control group, the EGCG group showed: ↑ Compressive resistance of decellularized porcine cartilage ↑ Fixation on the decellularized porcine cartilage coefficient of friction against glass ↑ Strength of cartilage–bone interference in shear ↑ Mechanical properties
Heinecke, 2010, [66]	**Model**: Equine primary articular cartilage **Treatments**: Pretreated with EGCG (4, 40, or 400 ng/mL) for 24 h and then cotreated with IL-1β (10 ng/mL) and TNF-α (1 ng/mL) for 24 h	Compared to the control group, the EGCG groups showed: ↓ COX-2 expression and PGE2 production ↓ NF-κB translocation to the nucleus
Huang, 2010, [64]	**Model**: Human primary osteoarthritic synovial adherent cells **Treatments**: Pretreated with EGCG (10, 20, or 50 μM) for 12 h and then cotreated with IL-1β for 12 h	Compared to the control group, the EGCG groups showed: ↓ COX-2 upregulation ↓ PGE2 and IL-8 production ↓ Phosphorylation of IKKβ
Huang, 2015, [74]	**Model**: Primary rabbit articular chondrocytes **Treatments**: Control, EGCG (5–20 µM) for 2, 4, and 6 days	Compared to the control group, the EGCG groups showed: ↑ Chondrocyte growth and synthesis of the cartilage extracellular matrix ↑ Expression of aggrecan, collagen II, and SOX9 genes ↓ Gene expression of collagen I ↓ Dedifferentiation of chondrocytes
Jin, 2020, [62]	**Model**: Primary porcine 3D encapsulated articular chondrocytes stimulated with IL-1β (10 ng/mL) **Treatments**: Control (no HA), EGCG (50 µM) + 2%HA, EGCG + 5%HA	Compared to the no HA group, the EGCG + 5%HA group showed: ↓ Expression of proinflammatory genes (COX2, TNF-α, MMP1, MMP13, ADMTS5) ↑ Chondrogenic regeneration via ↑ gene expression of COL2, SOX9, ACAN
Rasheed, 2009, [73]	**Model**: Human primary chondrocytes stimulated with ACE for 8 h **Treatments**: Pretreated with EGCG (25, 75, or 150 µM) for 2 h	Compared to the control group, the EGCG groups showed: ↓ Gene expression and production of TNF-α and MMP-13 via ↓ p38-MAPK and JNK activation ↓ IκBα protein degradation in the cytoplasm, followed via ↓ activation and translocation of NF-κB to the nucleus
Rasheed, 2016, [67]	**Model**: Human primary chondrocytes with and without IL-1β (5 ng/mL) Transfected with miRNA inhibitors **Treatments**: Control, IL-1β, IL-1β + EGCG (20–50 μM) for 24 h	Compared to the IL-1β group, the IL-1β + EGCG groups showed: ↓ COX-2 expression, PGE2 production via ↑ hsa-miRNA-199a-3p expression
Rasheed, 2018, [87]	**Model**: Human primary chondrocytes with and without IL-1β (5 ng/mL) **Treatments**: Control, IL-1β, IL-1β + EGCG (20–50 μM) for 24 h	Compared to the IL-1β group, the IL-1β + EGCG groups showed: ↑ Expression 19 miRNAs, ↓ 17 miRNAs, and no change in 1311 miRNAs ↓ ADAMTS5 expression ↑ hsa-miRNA-140-3p expression
Singh, 2002, [69]	**Model**: Human primary chondrocytes cotreated with IL-1β (2 ng/mL) **Treatments**: Control, EGCG (1, 10, 50, or 100 μM) for 12 or 24 h	Compared to the control group, the EGCG groups showed: ↓ iNOS expression and NO production ↓ IκBα protein degradation in the cytoplasm, followed by activation and translocation of NF-κB to the nucleus
Singh, 2003, [70]	**Model**: Human primary chondrocytes cotreated with IL-1β (2 ng/mL) **Treatments**: Control, EGCG (100 µM) for 30 min	Compared to the control group, the EGCG groups showed: ↓ Phosphorylation of JNK isoforms ↓ Accumulation of hosphor-c-Jun and DNA binding activity of AP-1 ↔ Activation of extracellular signal-regulated kinase p44/p42 (ERKp44/p42) or p38-MAPK
Zhang, 2019, [71]	**Model**: LPS-stimulated ATDC5 cells to mimic an inflammatory response during OA **Treatments**: Control, pre-GTP (100 µg/mL) for 24 hour, LPS, and GTP + LPS	Compared to the LPS group, the GTP + LPS group showed: ↓ Cell damage ↓ MAPK and NF-κB cascades via positively regulating miRNA-9
Zheng, 2019, [75]	**Model**: Human fibroblast-like synoviocyte-OA cells **Treatments**: EGCG (4 mg/mL), GA, EGCG + GA, EGCG + GA-NPs for 72 h	Compared to the EGCG group, the EGCG + GA-NPs group showed: ↓ Arthritic activity
***Animal Studies***		
Haqqi, 1999, [88]	**Model**: A mouse model of collagen-induced arthritis **Treatments**: Control, GTP (0.2% in water) for 7 weeks	Compared to the control group, the GTP group showed: ↓ Arthritic incidence ↓ Total IgG and type II collagen-specific IgG levels in serum and arthritic joints ↓ Expression of proinflammatory genes (COX2, TNF-α, IFN-γ) in arthritic joints ↓ Joint infiltration by TNF-α and IFN-γ-producing cells in arthritic joints ↑ Neutral endopeptidase activity in arthritic joints
Jin, 2020, [62]	**Model**: A rat model of surgically induced OA **Treatments**: Control (no HA), EGCG + 5%HA for 3 weeks	Compared to the no HA group, the EGCG + HA group showed: ↓ Cartilage loss (GAG, type II collagen) ↓ Expression of collagen I and X
Leong, 2014, [90]	**Model**: A mouse post-traumatic OA model using the destabilization of the medial meniscus (DMM) Male C57BL/6 mice (5–6 months old) **Treatments**: Sham, sham + EGCG (25 mg/kg), DMN, DMN + EGCG (25 mg/kg) for 4 and 8 weeks	Compared to the DMN group, the DMN + EGCG group showed: ↓ Progression in early and midstage OA as shown by decreased Safranin O staining and OARSI scores ↓ OA-associated pain symptoms as shown by higher locomotor behavior (distance traveled) ↓ Degradation of both type II collagen and aggrecan in the articular cartilage matrix ↓ Staining of MMP-13 and ADAMTS5 in the articular cartilage ↓ Gene expression of MMP-1, MMP-3, MMP-8, MMP-13, ADAMTS5, IL-1β, and TNF-α in the articular cartilage ↑ Gene expression of MMP-repressing transcriptional regulator CITED2 in the articular cartilage ↓ CCR2 and IL-1β and TNF-α in the DRG
Sobhi, 2007, [89]	**Model**: A mouse model of intra-articular carrageenan-induced OA Rats (150–200 g) **Treatments**: Control, OA, OA + GTE (1.5% *w/v*) for 3 weeks	Compared to the OA group, the OA + GTE group showed: ↓ Plasma lipid peroxides, NO, and total thiols ↓ Numbers of inflammatory cells infiltrating the synovial membrane at arthritic joint ↓ Erosive necrosis, chondrodysplasia, congestion, edema, mineralization, and multinucleated giant cells infiltration No cartilage and bone erosion
***Human Studies***		
Hashempur, 2018, [92]	**Model**: Randomized open-label active-controlled clinical trial Adults (*n* = 55) with knee OA **Treatments**: Control group (diclofenac at 100 mg/d) and intervention group (GTE at 1500 mg/d plus diclofenac) for 4 weeks	Compared to the control group, the intervention group showed: ↓ VAS pain, total WOMAC, WOMAC-physical function scores ↔WOMAC-pain, WOMAC-stiffness
Takiguchi, 2019, [18]	**Model**: 5-year cohort study (*n* = 11,091) (age range 40–72 years) with no history of knee OA	In men only, but not in women, older age, higher BMI, higher METs score, less smoking, and lower green tea consumption were associated with incident knee OA

ACAN, aggrecan; ACE, AGE, advanced glycation end products; AP-1, apolipoprotein-1; CCR2, C-C Motif Chemokine Receptor 2; CIA, collagen-induced arthritis; CITED2, Cbp/p300-interacting transactivator 2; COL2, collagen type II; COX, cyclooxygenase; DRG, dorsal root ganglion; EGCG, epigallocatechin gallate; ENA-78, epithelial neutrophil activating peptide-78; ERK, extracellular signal-regulated kinases; GA, glucosamine; GAG, glycosaminoglycan; GCP-2, granulocyte chemotactic protein-2; GM-CSF, granulocyte macrophage colony stimulating factor; GRO, growth-related oncogene; GTE, green tea extract; GTP, green tea polyphenols; HA, hyaluronic acid; IFN-γ, interferon-γ; iNOS, inducible nitric oxide synthase; IL, interleukin-β; IP-10, interferon-gamma-inducible protein-10; JNK, c-jun *N*-terminal kinase; LDH, lactate dehydrogenase; LIF, leukemia inhibitory factor; LPS, lipopolysaccharide; MAPK, mitogen-activating protein kinases; MMP, matrix metalloproteinases; NAP-2, nucleosome assembly protein-2; NF-κB, nuclear factor kappa-B; NO, nitric oxide; NP, nanoparticles; OA, osteoarthritis; OARSI, osteoarthritis research society international; PGE2, prostaglandin E2; SOX9, SRY-Box Transcription Factor 9; TGF-β, transforming growth factor-β; TGF-βRII, transforming growth factor-β receptor-II; TNF-α, tumor necrosis factor-α; VAS, visual analogue scale; WOMAC, Western Ontario and McMaster Universities Osteoarthritis Index. ↑, increase; ↓, decrease; ↔, no change.

**Table 3:** Summary of effects of EGCG on selected miRNA expressions of cells.

First Author, Year of Study, Citation/Cell Type	Effect of EGCG on Selected miRNA Expression
Rasheed, 2018 [87], human osteoarthritic chondrocytes	↑ *Upregulated expression of the following miRNA:* hsa-let-7a-5p; hsa-let-7b-5p; hsa-let-7c; hsa-let-7d-5p; hsa-let-7f-5p; hsa-let-7i-5p; hsa-miR-100-5p; hsa-miR-140-3p; hsa-miR-193a-3p; hsa-miR-199a-3p; hsa-miR-27b-3p; hsa-miR-29a-3p; hsa-miR-320b; hsa-miR-34a-5p; hsa-miR-3960; hsa-miR-4284; hsa-miR-4454; hsa-miR-497-5p; hsa-miR-5100 ↓ *Downregulated expression of the following miRNA:* hsa-let-7e-5p; hsa-miR-103a-3p; hsa-miR-125b-5p; hsa-miR-151a-5p; hsa-miR-195-5p; hsa-miR-222-3p; hsa-miR-23a-3p; hsa-miR-23b-3p; hsa-miR-26a-5p; hsa-miR-27a-3p; hsa-miR-29b-3p; hsa-miR-3195; hsa-miR-3651; hsa-miR-4281; hsa-miR-4459; hsa-miR-4516; hsa-miR-762
Zhang, 2019, [71], murine chondrogenic ATDC5 cells	↑ Upregulated expression of miRNA-9
Rasheed, 2016, [67], human osteoarthritic chondrocytes	↑ Upregulated expression of hsa-miR-199a-3p

↑, increase; ↓, decrease.

**Table 4:** Summary of studies on the effects of green tea and EGCG on skeletal muscle health.

First Author, Year, Citation	Experimental Design and Treatments	Effects of Green Tea or EGCG
***Cellular Studies***		
Babu, 2017, [95]	**Model**: C2C12 skeletal muscle cells **Treatment**: Pretreated with or without GTE (20, 40, and 80 μg/mL) for 2 h followed by with or without citrinin treatment (0, 25, 50, 75, and 100 μM) for 24 h	Compared to citrinin the treated without GTE group, the citrinin-treated GTE group showed: ↑ Cell viability dose-dependently ↓ Citrinin-induced LDH release ↑ Myotube integrity and cell morphology ↓ ROS 3-fold at 80 μg/mL ↓ Citrinin-induced antioxidant enzyme activity
Dorchies, 2009, [94]	**Model**: Primary culture muscle cells from an mdx mouse model treated with H_2_O_2_ **Treatment**: Control (no EGCG) or EGCG (10 mg/mL) for 48 h	Compared to the control group, the EGCG group showed: ↑ Glutathione content ↑ Cell survival ↑ 67LR (a receptor for EGCG)
Hong, 2020, [137]	**Model**: C2C12 skeletal muscle cells with or without 100 μM of H_2_O_2_ for 48 h **Treatment**: Control (no EGCG), GTE or tannase-converted GTE (1, 5, 10, 15, and 20 μg/mL) for 24 h	Compared to the control group, the H_2_O_2_-treated tannase-converted GTE group showed: ↑ Myotube density and fusion ↑ mRNA expression of myogenin, Myf5, MyoD ↑ FOXO1 and FOXO3 transcription levels ↓ Oxidative-stress-induced ↓ AMPK
Kim, 2017, [139]	**Model**: Satellite cells isolated from 8-week-old C57BL/6 mice **Treatment**: Control (no EGCG) and EGCG (10 μM) for 72 h	Compared to the control group, the EGCG group showed: ↑ MHC (myogenic factor) ↑ TAZ localization TAZ knockdown inhibits EGCG-induced myogenic differentiation
Kim, 2017, [138]	**Model**: C2C12 myoblasts **Treatment**: Control (no EGCG) and EGCG (10 μM) for 24 h	Compared to the control group, the EGCG group showed: ↑ MHC content ↑ Myogenin and muscle creatine kinase gene expression ↑ Number of multinucleated cells
Mirza, 2014, [121]	**Model**: C2C12 skeletal muscle cells with or without starvation media and TNF-α **Treatment**: Control (no EGCG) or EGCG (10–150 μM) for 24 h	Compared to the control group, the EGCG group showed: ↓ Protein degradation at 10 μM ↑ Protein degradation at >10 μM ↑ pAkt and pFoxO3a expression at >10 μM ↔ pAkt and pFoxO3a expression at 10 μM
Mirza, 2016, [144]	**Model**: C2C12 skeletal muscle cells treated with or without PIF (4.2 nM) or TNF-α (25 ng/mL) **Treatment**: Control (no EGCG) and EGCG (10 μM) for 24 h	Compared to the control group, the protein-degradation-induced EGCG group showed: ↓ PIF- or TNF-α-induced protein degradation ↔ Protein synthesis rates
Wang, 2016, [93]	**Model**: C2C12 skeletal muscle cells **Treatment**: Control (no EGCG) or EGCG (0, 5, 25, and 50 μM) with or without H2O2 (300μM) for 48 h	Compared to the control group, the EGCG group (at 25 μM) showed: ↓ SDH ↑ CAT and GSH-Px activity ↓ PGC-1α and NRF-1 protein content ↓ Intracellular ROS levels Compared to the EGCG group, the EGCG and H_2_O_2_ groups showed: ↑ p-AMPKα/AMPKα ↑ MyHC 1
***Animal Studies***		
Alway, 2014, [143]	**Model**: Brown Norway inbred aged (34 months) rats **Treatment**: Fed a control diet (no GTE) or GTE (50 mg/kg) for 7 days prior and throughout. Rats either received 14 days hindlimb suspension or 14 days hindlimb suspension and 14 days ambulation	Compared to the control group, the GTE with ambulation group showed: ↑ Activation of myogenic progenitor cells ↑ Muscle fiber area ↓ Bax and FADD
Alway, 2015, [140]	**Model**: Fischer 344 brown Norway aged (34 months) rats **Treatment**: Fed control (no GTE) or GTE (50 mg/kg) for 7 days prior and throughout. Rats either received 14 days hindlimb suspension or 14 days hindlimb suspension and 14 days ambulation	Compared to the control group or GTE with suspension group, the GTE with suspension and ambulation group showed: ↓ Suspension-induced muscle mass loss, cross-sectional area, and tetanic force ↔ Muscle mass and force during ambulation ↑ Number of nuclei adjacent to basal lamina (SC proliferation) ↑ Number of nuclei inside sarcolemma (SC differentiation) ↓ Protein carbonyl levels
Bhattacharya, 2015, [142]	**Model**: Young adult male BALB/c mice **Treatment**: Fed a control diet (no EGCG) or EGCG (250 mg/kg/d) for 39 days and given access to a running wheel	Compared to the control group, the EGCG group showed: ↑ Lean body mass (trend)
Buetler, 2002, [99]	**Model**: Mdx mouse treated with or without *tert*-butylhydroperoxide **Treatment**: Fed standard diet supplemented with or without 0.01% and 0.05% (by wt) GTE for 4 weeks	Compared to the control group, the GTE group showed: ↓ Necrosis in the fast-twitch muscle elongator digitorum longus ↓ Oxidative stress induced by *tert*-butylhydroperoxide treatment
Call, 2008, [132]	**Model**: C57BL/6J mice treated with a high-fat diet **Treatment**: Fed a control diet (no GTE) or GTE (0.5%, 45% EGCG) and endurance exercised (voluntary wheel running) for 3 weeks	Compared to the control group, the GTE group showed: ↑ Total distance running (128%) ↑ Citrate synthase activity ↓ Serum creatine kinase
Cao, 2007, [110]	**Model**: Male Wistar Rat **Treatment**: Rats were fed a high-fructose diet (induced insulin resistance and oxidative stress) and green tea solid extract (EGCG 12.75%) (1 or 2 g/kg) diet for 6 weeks	Compared to the control (high-fat alone), the GTE group showed: ↑ mRNA expression of anti-inflammatory tristetraproline family in liver and muscle ↓ mRNA expression of proinflammatory genes in liver and muscle
Chen, 2020, [145]	**Model**: Male BALB/c mice **Treatment**: Fed a control diet (no GTE) or GTE (0.2 g/kg) and/or endurance exercised (treadmill running) for 8 weeks **Model**: C2C12 skeletal muscle cells **Treatment**: Pretreated with or without (control group) GTE (0.01%) and then exposed to ammonium chloride (5 mM)	Compared to the control group, the GTE with exercise group showed: ↑ Endurance capacity and urea-cycle-related gene expression Compared to the control group, the GTE group showed: ↓ Hyperammonemia-induced reduced mitochondrial respiration
Dorchies, 2006, [98]	**Model**: Mdx mice **Treatment**: Fed a standard diet with or without 0.25% GTE (EGCG, 0.1%) for 5 weeks	Compared to the control group, the GTE group showed: ↑ Phasic and tetanic tensions almost to matched control values ↑ Residual force by 30–50%
Evans, 2010, [114]	**Model**: Mdx mice **Treatment**: Fed a control diet (no GTE), 0.25%, or 0.5% GTE (EGCG >45%) for 21 days	Compared to the control group, the GTE group showed: ↓ Serum creatine kinase ↑ Area of normal fiber morphology ↓ Area of regenerating fibers ↓ NF-κB staining in regenerating muscle fibers
Haramizu, 2013, [112]	**Model**: Male ICR mice **Treatment**: Fed a control diet (no GTE) or GTE (0.5%) for 3 weeks. Mice were then downhill running exercised (induce muscle damage)	Compared to the control group, the GTE group showed: ↓ Muscle-damage-induced reduction in voluntary wheel-running activity, tetanic force ↓ Muscle-damage-induced reduction in plasma creatine phosphokinase levels ↓ TNF-α, IL-1β, MCP-1
Hong, 2020, [104]	**Model**: Male ICR aged mice (24 months) **Treatment**: Fed a control diet, epicatechin (2 mg/kg), or tannase-converted GTE (20 or 40 mg/kg) diet	Compared to the control group, the tannase-converted GTE group or epicatechin group showed: ↑ Lean mass at 40 mg/kg for the tannase-converted GTE group ↑ MyoD, myogenin for both groups ↓ Myostatin for both groups ↑ S6K, follistatin at 40 mg/kg for the tannase-converted GTE group ↑ SOD, CAT for both groups ↓ FoxO3a, ↑ mTOR at 40 mg/kg for the tannase-converted GTE group ↓ MuRF-1 and atrogin-1 for the tannase-converted GTE group
Kumaran, 2008, [100]	**Model**: Male albino Wistar aged (34 months) rats **Treatment**: Fed a control diet (no EGCG) or EGCG (100 mg/kg/d) for 30 days	Compared to the control group, the EGCG group showed: ↓ Lipid peroxidation and protein carbonyl content
Meador, 2015, [127]	**Model**: Sprague–Dawley aged (20 months) rats **Treatment**: Fed a control diet (no EGCG) or EGCG (200 mg/kg) for 8 weeks	Compared to the control group, the EGCG group showed: ↑ Muscle mass, cross-sectional area ↑ IL-15, IGF-1
Murase, 2005, [130]	**Model**: BALB/c mice **Treatment**: Fed a control diet (no GTE) or GTE (0.2–0.5%) for 10 weeks and then endurance exercised (swimming until exhaustion)	Compared to the control group, the GTE group showed: ↑ Swimming times to exhaustion (8–24%) ↑ β-oxidation activity, fat oxidation, and plasma free fatty acid concentration ↓ Respiratory quotient and plasma lactate concentration ↑ Fatty acid translocase/CD36 mRNA expression
Murase, 2006, [131]	**Model**: BALB/c mice **Treatment**: Exercised with or without GTE (0.2–0.5%) for 10 weeks and then endurance exercised (treadmill running until exhaustion)	Compared to the control (exercise only) group, the GTE group showed: ↑ Running times to exhaustion 30% ↓ Respiratory exchange ratio, malonyl-CoA content, and plasma lactate concentration ↑ β-oxidation activity, muscle glycogen content, and plasma free fatty acid concentration
Murase, 2008, [129]	**Model**: Senescence-accelerated prone mice **Treatment**: Fed a control diet (no GTE) or GTE (0.35%, EGCG 41%) for 10 weeks and/or endurance exercised (treadmill running)	Compared to the control group, the GTE group showed: Maintained endurance capacity ↓ Oxidative stress Compared to the control group, the GTE with the exercise group showed: ↑ Oxygen consumption, fatty acid β-oxidation, mitochondria-related mRNA expression
Nakae, 2008, [97]	**Model**: Mdx mice **Treatment**: Fed vehicle (control group) or EGCG (180 mg/kg/d) for 5 weeks	Compared to the control group, the EGCG group showed: ↓ Development of dystrophic muscle lesions
Onishi, 2018, [141]	**Model**: Senescence-accelerated mouse prone-8 **Treatment**: Fed a control diet, an HFD diet, or HFD with 0.5% GTEs (71.68% EGCG) (GTE group) diet for 4 months	Compared to the HFD group, the GTE group showed: ↑ Muscle mass ↑ Akt and S6K phosphorylation
Ota, 2011, [101]	**Model**: BALB/c mice **Treatment**: Fed a control diet (no GTE) or a diet containing 0.5% GTE (81% catechins) for 14 days, and then mice were subjected to continuous tail suspension for 10 days	Compared to the control group, the GTE group showed: ↓ Unloading induced muscle tetanic force loss ↑ Total antioxidant potential ↓ Carbonylated protein levels
Sae-Tan, 2014, [128]	**Model**: C57bl/6J mice treated with a high-fat diet **Treatment**: Fed control (high-fat) diet or 0.32% EGCG for 16 weeks	Compared to the GTE without exercise group, the GTE with exercise group showed: ↓ Body mass and visceral fat ↑ PGC-1α, Cytb, CO III (mitochondrial metabolism enzymes)
Serrano, 2013, [124]	**Model**: Male C57BL6 mice **Treatment**: Fed a control diet or tea beverage (EGCG 37.8%) for 3 months	Compared to the control group, the EGCG group showed: ↓ Oxidative damage ↑ AMP-activated protein kinase α levels ↓ UCP-2 and UCP-4 ↑ Porin ↓ Mitochondrial DNA to nuclear DNA ratio
Shen, 2012, [102]	**Model**: Female Sprague–Dawley rats **Treatment**: Fed a high-fat diet for 4 months and then fed with or without GTE (0.5%) for additional 4 months	Compared to the control (high-fat without GTE) group, the GTE group showed: ↑ Glutathione peroxidase (reduces ROS)
Takahashi, 2017, [45]	**Model**: Brown Norway inbred aged (34 months) rats **Treatment**: Fed a control diet (no EGCG) or EGCG (50 mg/kg). Rats either received 14 days hindlimb suspension or 14 days hindlimb suspension and 14 days ambulation	Compared to the control group, the EGCG without ambulation group showed: ↑ ATG16L2, SNCA, TM9SF1, Pink1, PIM-2 gene expression ↑ ATG12, ↓ Beclin-1 protein content Compared to the control group, the EGCG group with ambulation showed: ↓ Beclin1 and LC3-II/I protein content
Wang, 2015, [103]	**Model**: Male Kunming mice **Treatment**: Intraperitoneally injected daily vehicle (control group) or EGCG at the dose of 55, 75, 100, and 200 mg/kg for 5 consecutive days	Compared to the control group, the EGCG group showed: ↓ SOD, catalase, glutathione peroxidase at 75 mg/kg ↔ Endogenous antioxidant activity Hepatotoxicity triggered at 200 mg/kg
Wang (2011), [113]	**Model**: Male C57BL/6 mice **Treatment**: Mice were injected with LLC cells (induced LLC tumor) and then fed EGCG (0.2 mg/mouse/d, prevention or 0.6, treatment mg/mouse/d) for 12 days	Compared to the control group, the EGCG group showed: ↓ Leukocyte infiltration ↓ NF-κB, and MuRF1, MAFbx (E3-ligases) ↓ Tumor volume and mass
Zhang (2020), [111]	**Model**: C57BL/6J mice **Treatment**:Mice were fed with HFD for 10 weeks to induce obesity. Obese mice were fed with continuous HFD, HFD with GTE (EGCG 12.5%), HFD with Ex, and HFD with both GTE and Ex for 8 weeks	Compared to the control group, the GTE + EX showed: ↓ blood glucose, serum total cholesterol, triglyceride, insulin, and alanine aminotransferase activity GTE, GTE + EX, EX ↓ proinflammatory gene expression GTE, GTE + EX ↑ IkBα (NF-κB inhibitor) activity GTE + EX ↑ glucose transport genes mRNA
***Human Studies***		
Hadi, 2017, [105]	**Model**: Men (*n* = 18; 20.94 ± 1.43 years) soccer players **Treatment**: Supplemented with or without (placebo group) GTE (450 mg/d) for 6 weeks **Exercise**: Maintained the same exercise schedule throughout the study	Compared to the placebo group, the GTE group showed: ↓ MDA levels
Jowko, 2015, [106]	**Model**: Male (*n* = 16; 21.6 ± 1.5 years) sprinters **Treatment**: Supplemented with or without (placebo group) GTE (980 mg/d) for 4 weeks **Exercise**: Repeated cycle sprint test	Compared to the placebo group, the GTE group showed: ↓ MDA and SOD levels ↑ Total antioxidant capacity
Panza, 2008, [107]	**Model**: Weight-trained men (*n* = 14; 19–30 years) **Treatment**: Supplement with water (placebo) or with GTE (200 mL) 3 times per day for 8 days. **Exercise**: Resistance exercise (Ex) protocol consisted of a warm-up followed by 4 sets of 10, 8, 6, and 4 repetitions, with 75%, 80%, 85%, and 90% of 1-RM, respectively	Compared to the placebo group, the GTE group showed: ↓ LH, aspartate aminotransferase, and uric acid ↑ GSH-Px ↓ Ex-induced increase in CK and xanthine oxidase
Silva, 2018, [109]	**Model**: Untrained men (*n* = 20; 23 ± 5 years) **Treatment**: Supplemented with or without (placebo group) GTE (500 mg/d) for 15 days and exercised (induced DOMS in triceps sural) before and after supplementation **Exercise**: Calf raising exercise trial one (maximal voluntary repetitions) and trial two (75% of maximal repetitions with repeated sets until >50% maximal of maximal repetitions could be achieved)	Compared to the placebo group, the GTE group showed: ↓ Creatine kinase
Townsend, 2018, [120]	**Model**: Untrained men (*n* = 38; 18–35 years) **Treatment**: Supplemented with, without (control group), or with placebo, aqueous proprietary polyphenol blend (40% catechins, 3–8% EGCG) for 28 days. Then subjects completed acute low body resistance exercise protocol (induced muscle damage) **Exercise**: Squat (6 sets of 10 repetitions) and leg press, leg extension (4 sets of 10 repetitions) exercises all at 70% 1-RM	Compared to the control group, the EGCG group showed: ↓ Muscle-damage-induced increase in Bcl-2, BAD
Tsai, 2017, [134]	**Model**: Healthy men (*n* = 8; 22 ± 1 years) with regular recreational physical activities 3/week **Treatment**: Supplemented with or without (control group) GTE (500 mg/d) for 8 weeks and then performed cycling exercise **Exercise**: 60 min cycling exercise at 75% VO2max after a 5 min warm-up exercise (50 W), 24 h postsupplement	Compared to the control group, the GTE group showed: ↑ Exercise-induced muscle GLUT4 protein content ↑ Fat oxidation energy reliance
Kuo 2015, [108]	**Model**: Forty untrained men (age: 20 ± 1 years) **Treatment**: Assigned to placebo (control), GTE, endurance training (EX), or endurance training with GTE (GTE + EX). GTE groups orally took GTE (250 mg/day) for 4 weeks **Exercise**: 75% oxygen uptake reserve for three 20 min sessions per week	Compared to the control group, the EX and GTE + EX groups showed: ↑ Exhaustive run time ↑ Maximal oxygen uptake ↓ Serum creatine kinase, MDA levels

AMPK, 5’ AMP-activated protein kinase; ATG, autophagy-related protein; Akt, protein kinase B; BAD, Bcl-2 associated agonist of cell death; Bax, bcl-2-like protein 4; Bcl-2, B-cell lymphoma 2; CAT, catalase; CD36, cluster of differentiation 36; CO III, cytochrome c oxidase subunit III; Cytb, Cytochrome b; DOMS, delayed onset muscle soreness; EC, epicatechin; EGCG, epigallocatechin gallate; FADD, Fas-associated protein with death domain; FoxO, Forkhead box (Fox) O; GLUT4, glucose transporter type 4; GSH-Px, glutathione peroxidase; GTE, green tea extract; HFD, high-fat diet; HK, hexokinase; IGF-1, insulin-like growth factor 1; IL-15, interleukin-15; LC3, microtubule-associated protein 1A/1B-light chain 3; LDH, lactate dehydrogenase; 67LR, 67 laminin receptor; MDA, malondialdehyde; Mdx, Dystrophin-deficient mice; MHC, myosin heavy chain; mTOR, mammalian target of rapamycin; MuRF-1, muscle RING-finger protein-1; Myf5, myogenic factor 5; MyoD, myoblast determination protein 1; NF-κB, nuclear factor kappa-light-chain-enhancer of activated B cells; NRF-1, nuclear respiratory factor 1; PGC-1α, Pparg coactivator 1 alpha; PIF, proteolysis-inducing factor; PIM-2, proviral Integrations of Moloney virus 2; Pink1, PTEN-induced kinase 1; PPAR-γ, peroxisome proliferator-activated receptor gamma; S6K, ribosomal protein S6 kinase beta-1; SDH, succinate dehydrogenase; SNCA, Synuclein Alpha; SOD, superoxide dismutase; TAZ, transcriptional coactivator with PDZ-binding motif; TM9SF1, transmembrane 9 superfamily member 1; TNF-α, tumor necrosis factor-alpha; UCP, uncoupling protein. ↑, increase; ↓, decrease; ↔, no change.
